# Barriers to and facilitators of implementing complex workplace dietary interventions: process evaluation results of a cluster controlled trial

**DOI:** 10.1186/s12913-016-1413-7

**Published:** 2016-04-21

**Authors:** Sarah Fitzgerald, Fiona Geaney, Clare Kelly, Sheena McHugh, Ivan J. Perry

**Affiliations:** Department of Epidemiology and Public Health, University College Cork, 4th Floor, Western Gateway Building, Western Road, Cork, Ireland

**Keywords:** Process evaluation, Implementation, Facilitators, Barriers, Workplace dietary intervention

## Abstract

**Background:**

Ambiguity exists regarding the effectiveness of workplace dietary interventions. Rigorous process evaluation is vital to understand this uncertainty. This study was conducted as part of the Food Choice at Work trial which assessed the comparative effectiveness of a workplace environmental dietary modification intervention and an educational intervention both alone and in combination versus a control workplace. Effectiveness was assessed in terms of employees’ dietary intakes, nutrition knowledge and health status in four large manufacturing workplaces. The study aimed to examine barriers to and facilitators of implementing complex workplace interventions, from the perspectives of key workplace stakeholders and researchers involved in implementation.

**Methods:**

A detailed process evaluation monitored and evaluated intervention implementation. Interviews were conducted at baseline (27 interviews) and at 7–9 month follow-up (27 interviews) with a purposive sample of workplace stakeholders (managers and participating employees). Topic guides explored factors which facilitated or impeded implementation. Researchers involved in recruitment and data collection participated in focus groups at baseline and at 7–9 month follow-up to explore their perceptions of intervention implementation. Data were imported into NVivo software and analysed using a thematic framework approach.

**Results:**

Four major themes emerged; perceived benefits of participation, negotiation and flexibility of the implementation team, viability and intensity of interventions and workplace structures and cultures. The latter three themes either positively or negatively affected implementation, depending on context. The implementation team included managers involved in coordinating and delivering the interventions and the researchers who collected data and delivered intervention elements. Stakeholders’ perceptions of the benefits of participating, which facilitated implementation, included managers’ desire to improve company image and employees seeking health improvements. Other facilitators included stakeholder buy-in, organisational support and stakeholder cohesiveness with regards to the level of support provided to the intervention. Anticipation of employee resistance towards menu changes, workplace restructuring and target-driven workplace cultures impeded intervention implementation.

**Conclusions:**

Contextual factors such as workplace structures and cultures need to be considered in the implementation of future workplace dietary interventions. Negotiation and flexibility of key workplace stakeholders plays an integral role in overcoming the barriers of workplace cultures, structures and resistance to change.

**Trial registration:**

Current Controlled Trials: ISRCTN35108237. Date of registration: 02/07/2013

**Electronic supplementary material:**

The online version of this article (doi:10.1186/s12913-016-1413-7) contains supplementary material, which is available to authorized users.

## Background

The increasing prevalence of diet-related diseases is a major global public health problem. The growing burden on population health and unsustainable cost escalation is crippling healthcare systems worldwide [1–4]. The causal factors of diet-related diseases are inherently complex and require complex solutions [5]. Behavioural interventions aim to improve dietary behaviours and reduce the associated burden of diet-related diseases at a population-level [6–8]. The Medical Research Council (MRC) advocate the importance of combining the evaluation of outcomes and processes when evaluating complex interventions [7]. Process evaluations monitor and evaluate the fidelity of interventions and can provide an in-depth understanding of factors that lead to the success or failure of implementing complex interventions [7, 9–11].

The workplace has been identified as an important health promotion setting as individuals spend long periods of time in their work environments and it also allows targeted health promotion programmes reach specific population groups [2, 8, 12, 13]. The workplace provides access to a stable population in a controlled setting, making it conducive to the implementation of complex interventions [14]. However, uncertainty exists regarding the effectiveness of complex workplace dietary interventions. Previous interventions have demonstrated limited efficacy with small effect sizes [15–17]. Although, some studies have reported that workplace interventions can have moderate positive effects on dietary behaviour in terms of healthier food choices and increasing fruit and vegetable consumption [8, 16–20], significant uncertainty remains regarding the long-term effects on dietary behaviour, health status outcomes and cost-effectiveness [8, 17, 21]. These interventions failed to include detailed process evaluations but recommended that future workplace interventions should integrate rigorous qualitative and quantitative evaluation methods to explore reasons for ambiguous findings [15–18, 22].

Very few comprehensive process evaluations of workplace dietary interventions have been conducted. Furthermore, few studies explore the opinions of those directly involved in workplace dietary interventions either as a decision maker or a participant. The evidence base consists mainly of process evaluations that evaluate low-intensity workplace health promotion interventions or workplace stress interventions. By design, low-intensity workplace health promotion interventions tend to focus solely on information provision and fail to investigate the effects of environmental approaches, such as food modification [14]. In contrast, high-intensity interventions are complex in nature and typically consist of a number of different interacting components. These components can include both information provision and environmental approaches such as, food modification, restricting options and provision of real incentives (i.e. price discounts) [14, 23]. These complex high-intensity interventions are informed by empirical evidence and theories and have a multi-level approach where they are specifically developed to target all stakeholders within an organisation (e.g. employers, caterers, employees) [7].

The available evidence on process evaluation of low-intensity workplace interventions has focused mainly on the effectiveness of interventions rather than on why interventions succeed or fail [24, 25]. The limited available evidence indicates that contextual factors, particularly structural and organisational changes can greatly influence the implementation of workplace interventions [26–29]. Evidence further suggests that in order to successfully implement workplace healthy eating interventions, it is vital to secure engagement by the catering team. Securing this engagement requires the research team to provide substantial support and understanding to the catering team [23]. The complexities of the modern working environment including on-going structural changes and competing work projects have also been indicated as factors that can impede intervention implementation. In contrast, active involvement of managers in implementation, negotiation skills, consideration of workplace culture and assessing readiness for change can serve as facilitators of implementation [27]. It has also been suggested that ensuring there is transparency in the implementation plan regarding roles and responsibilities of each team member can help facilitate intervention implementation [11, 27]. Similarly, contextual factors were also identified as influential in the implementation of a health promotion intervention in four Danish industrial canteens and structural changes which resulted in downsizing, high employee turnover and job insecurity impeded successful implementation [29].

There are a number of change theories and frameworks which describe the implementation of interventions within organisations. These theories suggest that fully understanding processes of change within organisations is critical for the successful development and implementation of workplace health promotion initiatives [30, 31]. Lewin’s model of organisational change is one such theory and involves, unfreezing of current attitudes to change, implementing the new intervention and refreezing new attitudes and behaviour by supporting and reinforcing change [32, 33]. This theory suggests that assessing organisational readiness for change and minimising the restraining factors of tacit organisational cultures are central for successful implementation of interventions and for achieving sustained change [30–33]. Schein’s theory on organisational change further suggests that in order to embed change, the intervention needs to become part of the culture of the organisation [31]. The principles of these theories are reinforced in implementation frameworks which outline the enablers and barriers to successful implementation within organisations [34]. Stakeholder buy-in, organisational support, supportive organisational culture, monitoring and evaluation are defined as enablers of implementation. The external environment, resistance to change and vested interests are outlined as barriers to implementation within organisations [34].

To improve the implementation of complex, high-intensity workplace dietary interventions and achieve sustainable organisational change, it is imperative that factors which facilitate and impede the implementation process are identified by exploring the opinions of those directly involved [9]. The aim of this study was to define and explore the facilitators of and barriers to the implementation of complex, high-intensity workplace dietary interventions from the perspectives of key workplace stakeholders, participating employees and research assistants delivering the intervention.

## Methods

### Context

The current study was carried out as part of the Food Choice at Work (FCW) study, a cluster controlled trial conducted in four large manufacturing workplaces in Cork, Ireland. Details of the FCW study have been published elsewhere [21]. Briefly, the FCW study assessed the comparative effectiveness of a workplace environmental dietary modification intervention and an educational intervention both alone and in combination versus a control workplace on employees dietary behaviours, nutrition knowledge and health status. Changes in employees’ dietary intakes and health status (BMI, waist circumference and blood pressure) outcomes were measured at baseline, follow-up at 3–4 months and 7–9 months. As the focus of the FCW study was to implement a complex dietary intervention in an environment that could tolerate different interacting intervention components, workplaces were purposively selected and allocated interventions. Workplaces were deemed eligible if they were manufacturing workplaces who employed more than 250 employees, had a daily workplace canteen, located in Cork, represented on the Industrial Development Authority of Ireland (IDA) website and were able to commit to all components of the complex intervention for the duration of the study [21]. In order to ensure that the participating workplaces and employees were representative of the general Irish workforce, demographic variables of non-participating employees were examined.

In the control workplace, data was collected at baseline and at each stage of follow-up. Participants in this workplace were informed that they were involved in a university-led study to observe employees dietary behaviours. The second workplace received a nutrition education intervention which comprised of three elements; group presentations, individual nutrition consultations and the provision of detailed nutrition information (traffic light menu-labelling, posters, leaflets and emails). The third workplace received an environmental dietary modification intervention which consisted of five elements 1) menu modification (restriction of fat, saturated fat, sugar and salt), 2) increase in fibre, fruit and vegetables, c) price discounts for fresh fruit, d) strategic positioning of healthier alternatives and e) portion size control [21]. Table 1 outlines the allocation of the interventions. The intervention design was developed by the research team who had specific expertise in public health nutrition and dietetics and was advised by catering stakeholders (Catering Managers Association of Ireland (CMAI)). The research team collaborated with the workplace stakeholders (human resources (HR) and catering managers) to implement the FCW interventions within each individual workplace. Each workplace was assigned a research workplace leader who was based on-site and collaborated with the workplace stakeholders to co-ordinate data collection for rotating shift schedules and monitor adherence to the interventions. Implementation was monitored and evaluated in all four workplaces using a detailed process evaluation throughout the intervention period, analysing perspectives of management stakeholders, participating employees and research assistants. Steckler and Linnan’s conceptual framework guided the process evaluation and was based on the components of context, reach, dose delivered, dose received, fidelity and recruitment [9].

**Table 1:** Allocation of FCW interventions across the workplaces and description of interventions

Workplace	Intervention implemented	Description of interventions
Control (Food & beverage industry)	Control site	Monitored employees eating behaviours.
Education (Health industry)	Nutrition education intervention	Nutrition education consisted of three elements: 1) monthly group presentations, 2) individual nutrition consultations and 3) detailed nutrition information (shopping cards, posters, leaflets and emails), including the application of a healthy eating traffic light coding system to daily menus and vending machines. This displayed the number of calories and nutritional breakdown of the meal or food item.
Environmental (Automotive industry)	Environmental dietary modification intervention	Environmental dietary modification consisted of five elements: 1) restriction of fat, saturated fat, sugar and salt, 2) increase fibre, fruit and vegetables, 3) price discounts on whole fresh fruit, 4) strategic positioning of healthier alternatives and 5) portion size control.
Combined (IT industry)	Combined intervention	All the elements of the nutrition education intervention and the environmental dietary modification intervention were implemented.

### Participants

For the process evaluation, purposive sampling was used to recruit management stakeholders who were involved in the intervention either through initial consultation, decision-making or on-going collaboration with the researchers who collected data. Employees who participated in the intervention were selected using random number generation software. At baseline 27 face-to-face semi-structured interviews (13 managers and 14 employees) were conducted and 27 interviews (12 managers and 15 employees) were conducted post intervention implementation. Where feasible the same people were interviewed at follow-up stage, however this was dependent on availability of participants. Research assistants who conducted the interviews were involved in recruitment and data collection but were not known to the participants they interviewed. Table 2 outlines the characteristics of managers and employees who took part. Purposive sampling was used to recruit research assistants for the focus groups. All research assistants involved in the FCW study were invited to participate at baseline and at follow-up stage. Nine out of eleven research assistants took part at baseline and four out of six research assistants took part at follow-up. The reason for non-participation in the focus groups was the part-time availability of research assistants and there were fewer researchers employed at follow-up stage.

**Table 2:** Characteristics of baseline and follow-up interviews conducted with managers and employees

	Managers		Employees	
Workplace	Baseline	Follow-up at 7–9 months	Baseline	Follow-up at 7–9 months
Control	2 (Occupational health and administrative managers)	3 (Occupational health and HR managers)	4 (2 male and 2 female)	4 (2 male and 2 female)
Education	3 (Occupational health, HR and catering managers)	3 (Occupational health, HR and catering managers)	3 (2 female and 1 male)	4 (3 male and 1 female)
Environmental	4 (Managing director, HR and catering managers)	3 (Managing director, HR and catering managers)	4 (2 female and 2 male)	4 (2 male and 2 female)
Combined	4 (Occupational health and catering managers)	3 (Occupational health and catering managers)	3 (1 female and 2 male)	3 (1 male and 2 female)

For the interviews, individuals were contacted by email and follow-up telephone call when necessary. The focus group moderator emailed research assistants and invited them to participate. All participants provided written informed consent. Data were digitally recorded and transcribed verbatim. To preserve confidentiality, data were anonymised.

### Topic guides

A co-investigator involved in the FCW study developed semi-structured topic guides for the interviews and focus groups (Additional files 1, 2, 3, 4, 5 and 6). As previously outlined, Steckler and Linnan’s conceptual framework was used to guide the process evaluation plan. Thus, the topic guides were based on the six components of the framework; context, reach, dose delivered, dose received, fidelity and recruitment [9]. These topic guides were reviewed and refined by research assistants on the study. Pilot interviews that were conducted at baseline and at follow-up stage, overall study objectives, preliminary analysis of baseline data and researchers’ experience of intervention implementation further informed the topic guides. For the interviews, the topic guides were used to explore facilitators of and barriers to the implementation of the interventions from the perspective of management stakeholders and employees. For the focus groups, the topic guides were used to explore the experiences of the research assistants delivering a complex intervention in the workplace.

### Data collection

Semi-structured face-to-face interviews were conducted at baseline between February and April 2013 and at follow-up stage between April and July 2014. Interviews were conducted in the workplaces and lasted between 40 and 60 min. The baseline focus group was conducted in May 2013 and the follow-up focus group was conducted post intervention implementation in August 2014. These were hosted in University College Cork by an independent moderator and lasted for 1 h. An assistant moderator took observational notes. In the interviews and focus groups probes were used to initiate discussion when there was a pause and also to further explore points of interest.

### Analytical tools

The framework approach was used for analysis of data [9, 35]. This was considered appropriate as the process evaluation had pre-specified objectives while it also permitted the emergence of unexpected themes. Framework analysis is dynamic, allowing for change throughout the analytical process while its systematic nature provides transparency. This was beneficial as multiple researchers were involved in data collection, analysis and interpretation. The following steps were completed [9]:

1. *Familiarisation:* Three researchers (SF, FG and CK) conducted the interviews. Researchers became familiar with the data by re-reading transcripts, audio tapes, field notes and observational notes. Recurring themes and initial ideas were noted in an analytical memo.
2. *Identification of a thematic framework:* Four researchers (SF, SMH, FG and CK) undertook initial coding of a selection of transcripts (one management stakeholder and one employee participant). These were subject to inter-coder reliability as one of the researchers (SMH) was not involved in data collection. Open coding allowed for an inductive approach. The preliminary coding framework was developed by discussing the convergence and divergence of codes. The researchers redefined this framework for subsequent stages of coding.
3. *Indexing:* This stage involved the indexing of specific parts of the data to correspond to the emerging themes. Data was imported into NVivo software (QSR International Pty Ltd) for coding. The refined coding framework was systematically applied to the data and the main thematic categories and sub-categories were formed.
4. *Charting*: The coded data was further abstracted and synthesised during the charting process by two of the researchers. This involved arranging themes into illustrative charts based on headings included in the thematic framework.
5. *Mapping and interpretation:* The charts provided a schematic diagram of the process evaluation which guided data interpretation. Interpretations were checked and discussed by two researchers. The interpretation of the themes was guided by the specific objectives of the study and also by the unexpected themes that emerged during analysis.

## Results

### Major themes

Four major themes emerged; 1) perceived benefits of participation, 2) negotiation and flexibility of the implementation team, 3) viability and intensity of intervention and 4) individual workplace structures and cultures. Depending on context, the latter three themes were found to have both a positive and negative impact on implementation and are discussed as either facilitators or barriers. Findings are presented from the perspective of management stakeholders, employees and research assistants.

### Perceived benefits of participation

Both managers and employees highlighted the benefits of participating in the study. Managers had a desire to improve company image and foster employee loyalty while employees had a desire to improve their health. The perception of a long-term benefit rather than the benefit itself facilitated implementation in the short-term as it encouraged engagement and fostered buy-in. Verbatim examples of this theme are included in Table 3.

1. Concern with company image: Managers had a vested interest in ensuring successful implementation of the interventions as they had a strong desire to portray a positive company image to both industry and employees. Managers believed that participation in the study would be a means of achieving this objective. Managers wanted to depict an image of a progressive company both nationally and internationally in the manufacturing industry. This desire facilitated implementation as managers were supportive of the interventions and they facilitated access to employees by releasing them from work activities to attend study appointments. Managers felt involvement in a university-led study would be regarded as prestigious by other companies. They expressed pride in being ‘chosen’ to participate and believed that it created a sense of elitism in the manufacturing industry. According to some of the researchers who collected data, a concern with company image motivated workplace stakeholders to provide recruitment and implementation support.
2. Managers’ personal interest: In some workplaces key workplace stakeholders expressed a personal interest in maintaining a healthy lifestyle. Occupational health stakeholders in the control and combined workplaces had a professional background in nursing and had great interest in supporting initiatives that would enhance health consciousness in the workplace. Similarly in the education workplace, a HR stakeholder had professional training and interest in nutritional sciences. This interest was a driver for workplace participation and ensured that implementation of the interventions received organisational support.
3. Fostering employee loyalty: A desire to improve relations between employers and employees was a motivating factor for participation. Managers identified the study as an opportunity to improve relations with employees. In order to demonstrate their support for the study to employees, they released staff from work activities for appointments and provided resources for the study. They believed that driving health consciousness among employees would foster employee loyalty and boost morale within the workplace which could result in financial benefits for the company by reducing absenteeism and increasing productivity. It was anticipated that this could be achieved by managers promoting participation in elements such as the healthy-eating group presentations.
4. Health concerns among employees: The main reasons for employees participating included age concerns, individual health concerns (weight, cholesterol level, blood pressure, and digestive disorders) and lifestyle concerns. Older participating employees felt pressure to keep up with younger employees in their fast-paced working environments. Employees were seeking health improvements in an effort to curtail any negative effects of ageing and the need to ‘slow down’ their working pace. Employees appreciated the investment their employers made in the study as it provided them with a unique opportunity to have a nutritional consultation and a free health check-up during their working hours. It reassured employees that their employer concerns went beyond generating profit hence they felt obliged to participate.

**Table 3:** Theme of ‘perceived benefits of participation’ and verbatim examples

Theme	Verbatim Examples
Perceived benefits of participation	*1. Concern with company image: “We were one of the ones to be chosen, that’s a huge cannon feather in our cap you know we’re thrilled about that and you know again to promote the fact that it’s not everybody that was selected….we were chosen as a company for a particular reason and we’re honoured to be included”* (HR manager, Environmental site—follow-up stage).
	*2. Managers’ personal interest: “I would have been the person who pushed it to say ‘let’s go and do this, it’s an opportunity, yeah’…having dieticians on site, having access to all this expertise you know, and it is a great pile of health promotion going on in the background”* (Occupational health, Control site—follow up stage).
	*3.Fostering employee loyalty: “If you’re trying to convince employees that you’re interested and trying to engage with them, show them that you care about their health and well-being so that’s a good engagement tool”* (Occupational health, nutrition education site—baseline stage).
	*“If we can keep our employees healthy, they’ll be happier, they’ll produce better work, they’ll hit their efficiencies a lot better and they’re more likely to be in here”* (HR, nutrition education site—follow-up stage).
	*4. Health concerns among employees*: *“We don’t have the luxury in this modern day and age of getting to 54, in days of old you’d get to this age and you pull back a little, there’s young and progressive people coming up underneath you and they take the pressure and that, that doesn’t happen today. They are going to work people until they’re 65”* (Employee, nutrition education site—follow-up stage).

### Flexibility and negotiation

The researchers who collected data and were involved in coordination and delivery of intervention elements were adaptable to dynamic workplace environments which facilitated implementation. This flexibility enabled the researchers to successfully negotiate with workplace managers on degrees of change that were agreeable to all parties and ensured the study received organisational support. Verbatim examples of this theme are included in Table 4.

1. Flexibility: The flexibility and adaptability of the researchers manifested itself in a number of ways. To facilitate timely data collection, it was critical for the researchers to adapt to the structure and practices of each worksite. Researchers were required to schedule appointments that complemented rotating shift patterns. Similarly, monthly group nutrition presentations were delivered multiple times each day to also complement rotating shifts. Data collection often occurred during busy times on site such as ‘end of quarter’. On these occasions, employees frequently rescheduled appointments and researchers had to facilitate these late changes. At the outset, managers were concerned that the target-driven culture of manufacturing workplaces would not be suitable for implementing a study that requires employee interaction and significant logistical planning. However, researchers’ adaptability to changes facilitated implementation.
2. Negotiation: The researchers also perceived negotiation as central to successful implementation. It was necessary for the researchers to negotiate a level of change that was agreeable to mangers, caterers and the researchers themselves. In some instances this resulted in changes to the planned intervention components or the scale of change. Effective communication with managers was necessary to reach a compromise with regards to what intervention elements were implemented and to what degree they were implemented, particularly for the environmental modification intervention. For example, the proposed portion size restrictions were heavily negotiated between the researchers and catering staff with compromises being made by all parties. Willingness to change among catering staff and researcher negotiation skills facilitated compromises being reached.
  The researchers described how certain meals appeared to be non-negotiable in the environmental and combined workplaces. The cooked breakfast was part of the workplace culture and researchers found reaching an agreement on modifying this option challenging. A compromise was eventually reached on reducing the portion size of the cooked breakfast and cooking method was changed from frying to baking when possible. In this instance, workplace culture was identified as a barrier to full-scale implementation. Catering stakeholders anticipated employee resistance to change in response to changes being made to the breakfast options. This expectation persisted and impeded the implementation of some of the environmental modification elements.
3. High-level workplace management support: Due to the target-driven culture in the manufacturing industry, supervisors were reluctant to release production staff to attend appointments. A disruption on the production line could lead to knock-on effects for overall site-level efficiencies. However, supervisors were instructed by managers to adapt to the demands of the intervention for the duration of the study period. To ensure that catering staff adhered to the intervention elements, management needed to reinforce the commitment that the workplace had made to the study. This was particularly evident in the environmental and combined workplaces, where environmental modification elements were implemented and more negotiation was needed in these workplaces. Stakeholder cohesiveness with regards to organisational support was central to achieving successful implementation.

**Table 4:** Theme of ‘negotiation and flexibility’ and verbatim examples

Theme	Verbatim Examples
Negotiation and flexibility	*1. Flexibility: “You need to adapt and be understanding because schedules do change so you go in with your full schedule and you mightn’t get all of them or people last minute can’t make it and you’re getting annoyed when you’re there on site waiting but out on site things are changing constantly so you really have to adapt”.* (Researcher 2 - follow-up stage)
	*2. Negotiation: “Changing down to nearly half, we just couldn’t, there would be uproar…we did a taste test, we put three plates out one with what we serve now, one with what UCC wanted us to serve and something somewhere in the middle that we felt we could serve and get away with, that’s the way we made our choice”* (Occupational health, combined intervention site baseline stage).
	*“The breakfast option alright was something that you couldn’t change too much. I suppose from their side they were just afraid that there would be a lot of backlash from the employees and there at the front line then dealing with it”* (Researcher 2 - follow-up stage)
	*3. High-level workplace management support: “I found it very, very hard to get product builders released for their sessions. That was a huge struggle for me, it’s the team leaders and they’re all about their metrics, they want to have, net efficiencies, be on target”* (Occupational Health—nutrition education site—follow-up stage).

### Workplace structures and cultures

Individual workplace structures and cultures had an impact on implementation. In workplaces where senior management were actively involved in the study, it encouraged employee participation and secured more buy-in from production supervisors and team leaders. In the environmental workplace, the support of HR managers went beyond providing basic logistical support and HR contacts became involved in providing recruitment support. Organisational restructuring and a ‘traditional’ workplace culture had a negative effect on implementation. Verbatim examples of this theme are included in Table 5.

1. Stakeholder buy-in: Employees recognised the importance of receiving ‘buy-in’ from catering and management stakeholders in order for the intervention to be successfully implemented. This was also highlighted by the researchers who acknowledged their flexibility and willingness to change as a crucial facilitating factor. Enthusiasm of caterers towards the intervention further facilitated the progress of implementation. Support of the catering company in their workplace stemmed from caterers realising that involvement in the study could be a valuable learning opportunity and serve as a foundation on which to enhance the knowledge of the catering staff. Catering stakeholders anticipated that their involvement would impress the head office of their catering company as staff will have the opportunity to apply the knowledge and skills they gained on how to produce healthy menus after the study period and also in future interventions. This long term potential benefit garnered buy-in from catering stakeholders and facilitated intervention implementation as they were more invested in making the intervention a success in their workplace.
2. Production work: Both managers and employees perceived shift work to be a barrier to implementation. This was due to the logistical problems of arranging appointments for shift workers outside standard office hours. However, it emerged that it was the nature of production work rather than the shift cycles that impeded implementation.
3. Organisational restructuring: Conversely, a number of workplace factors were identified as aspects that impeded implementation. Two of the largest workplaces (education and combined) underwent major restructuring during the study. This involved the relocation of a large number of employees from both workplaces, which resulted in them being ineligible to participate in the study as they were no longer exposed to the intervention. As a direct result of the restructuring, a large proportion of the remaining employees changed shift patterns. In order to deal with these effects researchers had to liaise with management on how to best minimise loss to follow-up and had to adapt elements of the study to these changes. This involved researchers creating an appointment schedule to facilitate changes in shift work patterns to encourage employees to complete all stages of data collection. The time it took to liaise with management regarding restructuring changes had a direct impact on the timeline of the study. Adjusting to the restructuring changes and the delays in recruitment meant that data collection timelines had to re-evaluated, however getting approval from the management stakeholders for these readjustments proved to be very time consuming.
4. Workplace culture: According to the researchers involved in data collection, the workplace culture provided challenges during implementation. This manifested itself particularly in the environmental modification site, with the majority of employees described as having ‘traditional’ eating habits. The cooked breakfast menu options and side portion of chips were described as part of the tradition of the workplace. The expectation of poor uptake of the interventions made catering stakeholders reticent to agree to all modifications. Catering stakeholders were cautious when agreeing changes which resulted in the cooked breakfast menu option not being fully modified in the workplace. However, as previously mentioned researchers overcame this by reaching compromises on method of cooking, portion size and reducing the number of days that chips were available in the workplaces.

**Table 5:** Theme of ‘workplace structures and cultures’ and verbatim examples

Theme	Verbatim Examples
Workplace structures and cultures	*1. Stakeholder buy-in: “We had really good contacts with HR, they helped with recruitment, they helped schedule some participants…..that was probably the easiest site in terms of scheduling and recruiting…. if someone didn’t turn up all I had to do was go downstairs and tell one of the HR people and they would actually go and get the employee”* (Researcher 1 - follow-up stage).
	*2. Production work: “There’s a big, discrepancy between the support staff and the people who work on the line, in that the support staff have that freedom to, to go to these things”* (Occupational health, nutrition education site—follow-up stage).
	*3.Organisational restructuring: “Those who are in charge they’d have the overall influence because they’re the ones bringing in the stock and stuff, so they have to be behind it 100 %. Like if there was opposition from the management that could hinder it”* (Employee, nutrition education site—baseline stage).
	*“Many employees they left the company and were moved to other departments, so it was hard to get them back for the last stage of the study but we got agreement from the managers in order to allow us to complete the last stage”* (Researcher 3 - follow-up stage).
	*4.Workplace culture: “Well it’s another concern, its more rural here, people are a bit more conservative about their food, I mean we’ve been asked over the years for stuff like Panini’s, honestly, I’d give them a week and they just don’t go” (*Catering Manager, environmental site baseline stage).

### Viability and intensity of interventions

The design of the interventions also impacted how they were implemented. The sustainability of the interventions and the ability of workplaces to tailor the interventions to meet the needs of their workplace facilitated implementation. The anticipated employee resistance to change in response to the environmental modification impeded implementation of the interventions. The intensity of the interventions also affected implementation. The high-intensity intervention (combined intervention) was well received by employees. However, the low-intensity interventions (education and environmental) did not meet employee expectations which impeded implementation. Verbatim examples of this theme are included in Table 6. 

1. Sustainability of interventions: Intervention design had impact on implementation. At the outset, catering staff were apprehensive about implementing environmental modification elements as they anticipated it would cause a significant increase in workload. However, it transpired that any extra workload initially created dissipated once the intervention was in place and as a result the study was easier to maintain. Environmental modification elements became part of the normal catering routine within workplaces even after the study, with workplaces sustaining elements. Similarly, the environmental modification site maintained the healthy default menu options, increased the number of ‘chip free’ days per week in the workplace and removed free-flowing sugar and salt from the canteen. The catering staff in the combined intervention decided to keep elements that modified the nutritional quality of food in terms of fat, saturated fat, sugar and salt.
  However, there was a perception among the researchers that catering stakeholders in the combined workplace found the initial implementation of the intervention burdensome in terms of extra workload. Researchers suggested that this caused a delay in implementation at the outset which was overcome through negotiation of elements that were more feasible for the catering staff to implement.
2. Tailoring of interventions: The advantage of being able to tailor the intervention to address certain needs was also alluded to by the employees. An employee being able to ‘pick and choose’ to engage with certain elements was not an intended feature of the study design. This occurred naturally throughout the study as employees reported that different elements of the intervention worked for them, for example, some employees found the health eating chat table more beneficial to them compared to the monthly group nutrition presentations. Employees also appreciated that participation in the study was open to all employees in the workplace, regardless of job position. This inclusive study design which allowed employees to adapt elements to meet their own requirements was perceived as a key facilitating factor for implementation by employees and management stakeholders. The intervention created scope to positively impact all employees in terms of dietary behaviour, regardless of participation in the study with all employees being exposed to the intervention in the canteen.
3. Information at a glance: Employees outlined how the traffic light system enabled them to make informed decisions with regards to healthy or unhealthy menu options. It provided information at a glance in a fast-paced environment which was particularly helpful to production workers as their lunch times were very restrictive. This visibility of the intervention was described as a talking point among employees and they discussed their clinical measurements, progress and feedback with each other. Displays of nutritional information in the canteen and the daily email of healthy options were considered effective. The traffic lights created a social desirability response as employees were reluctant to choose a menu option that was coded as red when they were eating in a group. It also emerged that since the study finished in the workplaces, employees and catering stakeholders found the absence of intervention very noticeable, mainly the traffic light coding system and the nutritional information that was displayed in the canteen. The design of the intervention in terms of its inclusive and visible nature was perceived to be a key facilitator for successful implementation.
4. Employee resistance to change: The potential for employee ‘backlash’ in response to choice restriction impeded implementation. Caterers anticipated that the implementation of choice restriction may create a sense of perceived powerlessness amongst employees. They also anticipated employee ‘backlash’ in reaction to the introduction of chip free days and reduced portion size. Some of these concerns were both anticipated and realised concerns. The combined intervention workplace reported that employees’ resistance to change was largely in response to the removal of some of the unhealthy options on the menu. This impeded the implementation of the intervention slightly as caterers were reluctant to introduce a further chip free day that had been suggested during the negotiation with the researchers. However, catering stakeholders were determined to implement the agreed intervention elements to an extent they thought was feasible. The expectation of resistance to change was one of the main reasons cited for negotiating the degrees of change in the workplace. There was a perception among researchers that the ‘backlash’ was not as great as expected. Researchers suggested that any resistance that occurred was due to a small minority in the workplaces and the catering company were capable of dealing with it.
5. Intervention intensity: Catering stakeholders and employees in the education and environmental workplaces felt that the study lost momentum towards the end of the study period. The interventions implemented in the education and environmental workplaces were low intensity by design compared to the high intensity intervention that was implemented in the combined workplace. Employees and catering stakeholders in the education and environmental workplaces felt that the interventions would have benefited from more regular stages of data collection and suggested that more emphasis should be placed on physical measurements and weight loss to increase intervention intensity. The low intensity interventions delivered in these workplaces did not meet employee expectations. Employees felt that delays in data collection and long stages of follow-up resulted in a loss of interest and focus in the study.

**Table 6:** Theme of ‘viability and intensity of intervention design’ and verbatim examples

Theme	Verbatim Examples
Viability and intensity of intervention design	*1. Sustainability of interventions: “It was much easier than I thought it was going to be…I was a little bit scared at the start of all the changes that would have to be made, but actually it was fine, it was fine, it was all quite manageable”* (Catering manager, environmental site—follow-up stage).
	*2. Tailoring of interventions: “Even though the study is over it still continued, there was no dramatic okay that’s done go back to the old ways, pretty much there’s a lot of things that we kept on board”* (Catering manager, combined intervention site—follow-up stage).
	*3. Information at a glance: “People are in a hurry so it was a perfect situation where you were rushing in and out you could still see at a glance what your options were in terms of healthy choices” (Occupational health, nutrition education site—follow-up stage).*
	*4. Employee resistance to change: “The glazed loin of bacon, we took it off for 2 weeks and we had something like 300 common cards or something you know it’s like, ‘where is bacon’ because it would always be on a Monday or Tuesday”* (Catering manager, combined intervention site—follow—up stage).
	*“I suppose from their side they were just afraid that there would be a lot of backlash from the employees and there at the front line then dealing with it but to be fair when we spoke again with them there wasn’t too much backlash”* (Researcher 2 - follow-up stage).
	*5. Intervention intensity: “It’s not very regular, should I say and it’s not very intrusive, you know what I mean… it’s the idea of, you know, getting weighed in once a week and kind of like the competition type thing”* (Employee, environmental site—follow-up stage).

## Discussion

This study aimed to establish what factors facilitated or impeded implementation of complex workplace dietary interventions. Four principal themes emerged; perceived benefits of participation, negotiation and flexibility of the implementation team, viability and intensity of intervention design and workplace structures and cultures. Contextual factors were found to heavily influence implementation. Tacit workplace cultures including ‘traditional’ menu preferences and anticipated and realised resistance to change prevented full-scale implementation of the environmental intervention. The target-driven culture of manufacturing workplaces impeded implementation as the researchers involved in data collection experienced challenges in arranging appointments with employees. Our results suggest that manufacturing production work rather than restrictive shift cycles impeded implementation of a complex workplace dietary intervention. Organisational restructuring caused delays to the study timeline, attrition and disruptions to schedules. These barriers persisted throughout the study but were eased by the flexibility and negotiation skills of the researchers. The adaptability of the implementation team was a vital facilitator for implementation and helped accommodate the impact of extensive organisational restructuring.

Despite consensus in the literature that workplace dietary process evaluations should be conducted concurrently with evaluations of outcomes, the current evidence base is extremely limited [25]. However, findings from this study are consistent with process evaluations of other types of organisational interventions. The structural environment can act as a major barrier to implementation if it cannot tolerate the intervention that is being implemented [34]. Previous research indicates that contextual factors have significant influence on the implementation of workplace interventions. Complexities of the modern working environment including structural changes, competing projects, employee turnover and downsizing have all been outlined as potential barriers to implementation [27, 29]. Workplaces are dynamic environments and their contexts cannot be controlled. The flexibility and adaptability of the researchers were important factors that helped the study overcome contextual barriers [23].

The findings are consistent with research that suggests stakeholder buy-in and supportive organisational cultures facilitate implementation [23, 27, 29]. Managers perceived benefits and personal interest in the study fostered their buy-in and support which facilitated implementation. Stakeholder consultation and buy-in is critical for successful implementation [34]. The implementation team openly consulted with each other throughout recruitment, intervention allocation and intervention implementation. This consultation process was beneficial for the researchers collecting data and coordinating and delivering the intervention as they were able to assess the capacity and suitability of each workplace for particular intervention elements. The process also assisted in workplaces providing organisational support to the study. Supportive organisational structures and systems are a key enabler of successful implementation [34]. This study reported the presence of strong organisational support from one of the workplaces whereby the HR manager assisted in recruiting and scheduling of employees for their appointments which facilitated timely implementation.

Our findings are in line with Lewin’s theory of organisational change which suggests that sustained organisational change is achieved by workplaces achieving an appropriate balance between minimising restraining factors and promoting facilitating factors [31–33]. Tacit organisational cultures such as resistance to change and fragmented relationships between workplace stakeholders need to be managed. Resistance to change is a key barrier to achieving sustainable organisational change [34]. This stage can also be referred to as the ‘Unfreezing’ stage of the Lewin’s theory whereby organisations need to recognise the need to change the current situation [31]. This was achieved through initial consultations between the research team and workplace stakeholders. The second stage is referred to as the ‘Transition’ stage and involves the actual implementation of the intervention which should promote new behaviours, values or attitudes. This was achieved by implementing the FCW interventions and in order to overcome resistance, negotiation on degrees of change occurred during the implementation process. Restrictive factors can be overcome by key workplace stakeholders reinforcing the benefits of participation and by negotiation and compromise to minimise negative internal politics. This step can also be referred to as the ‘Re-freezing’ stage where the change becomes fixed in the workplace culture of the organisation. Schein’s theory of organisational change is also reflected in our results as such positive reinforcement and minimising of restrictive facts can help the change to become embedded in the workplace culture [31].

Based on the results of this study, it is vital that future intervention teams consider individual workplace cultures and structural changes during the development and implementation of interventions. The effects of structural changes need to be monitored regularly throughout the study. Workplaces need to be able to tailor the intervention to meet their own specific needs with minimal effort [11]. Consultation with key stakeholders should be an integral aspect of complex workplace interventions prior to implementation and can assist in considering the challenges of manufacturing work and in assessing an organisations readiness for change. Stakeholders need to be aware of the demands of the study and researchers need to determine if the workplace structure can tolerate all aspects of the intervention. Understanding the feasibility of implementing the FCW interventions will help researchers and workplace stakeholders anticipate future barriers of implementing multisite workplace dietary interventions.

Consideration also needs to be given to employee expectations. Employees’ expectations of an intervention can impact how it is implemented and received. The control, education and environmental workplaces received low intensity interventions and employees in these workplaces felt that the momentum of the study was lost over time. Employees had anticipated an interactive intervention that would be of high intensity with more frequent physical assessments. This perceived loss of momentum impeded implementation as employees’ interest in the study declined. As the employees were blinded to their interventions during the FCW trial, the researchers were unable to clarify the employees’ expectations of the different interventions. However, in practice, the authors agree that employees should be made fully aware of what the intervention entails at the outset.

This study has several strengths and limitations. To ensure rigour, Guba’s framework for ensuring trustworthiness in qualitative research was adhered to [36]. This framework proposes four criteria for assessing trustworthiness; credibility, transferability, dependability and confirmability. Credibility is concerned with assessing the internal validity of the findings, ensuring they are congruent with reality [36]. In an attempt to ensure credibility, well established research methods were used. These methods included the use of random sampling when appropriate, holding regular debriefing discussions during data collection and triangulating findings from different stakeholders. Transferability refers to the extent to which findings can be generalised or applied to other contexts [36]. These findings may be generalisable nationally and transferable internationally as the workplaces included are multi-national manufacturing companies with similar worldwide structures and operations. Dependability addresses the reliability of the study and whether or not the same results would be achieved if the study were repeated [36]. In this study dependability is concerned with the repeatability of the methods [36, 37]. Both an in-depth methodological description which reported extensively on processes used and a comprehensive description on how changing contexts affected the implementation of interventions were provided.

The fourth construct of confirmability is concerned with the objectivity of the research [36]. In this study, researcher bias cannot be ruled out as some of the authors were involved in the overall FCW study and were familiar with participants. Efforts were made to remain as objective as possible with researchers conducting interviews in workplaces that they did not visit for data collection. Furthermore, there were a number of members of the multidisciplinary FCW research team involved in the analysis and interpretation of findings. However, the inclusion of respondent validation may have been useful as respondents’ interpretation of emerging results can help refine findings and strengthen conclusions.

## Conclusion

The findings of this study can be used to support the argument that process evaluations should be carried out concurrently with effectiveness studies for workplace interventions [25]. This study demonstrates how process evaluations can be used to explore factors that may influence implementation in controlled intervention studies and highlights the complexities associated with implementing complex workplace dietary interventions. Perceived benefits of participation, stakeholder buy-in and organisational support are intrinsic facilitators of implementing workplace dietary interventions. Flexibility and negotiation play a pivotal role in overcoming the barriers of individual workplace cultures, structures and resistance to change. Interventions also need to be adaptable as the manufacturing companies need to tailor interventions to meet specific structural and cultural requirements of their workplaces. Workplace stakeholders play a central role in achieving organisational change by reinforcing benefits and providing fundamental organisational support. Cohesiveness between different stakeholders within the workplace and between the implementation team (stakeholders involved in co-ordination and delivery of interventions and researchers involved in data collection and delivery of intervention elements) is essential for successful implementation. Intervention implementation within organisations is largely influenced by contextual factors. To achieve organisational change, these factors need to be carefully considered prior to implementation along with an assessment of readiness for change. This study provides an in-depth understanding of the implementation context to further illuminate the findings of the FCW study. Our results may also inform the implementation of future workplace dietary interventions for the development of sustainable diet-related disease prevention and provide an opportunity for scaling of similar interventions for use in practice) for use in practice.

### Ethics

Ethical approval was granted by the Clinical Research Ethics Committee of the Cork Teaching Hospitals in Ireland, March 2013.

### Consent for publication

Written informed consent was provided by all participants.

### Availability of data and materials

Topic guides which were used in the interviews and focus groups are available as additional supporting files. However, signed confidentiality agreements prevent us from sharing transcripts.

## Additional files

Additional file 1: Topic Guide for Employees – Baseline stage. (DOCX 27 kb) Additional file 2: Topic Guide for Focus Group (Baseline stage). (DOC 31 kb) Additional file 3: Topic Guide for Managers (Baseline stage). (DOCX 23 kb) Additional file 4: Topic Guide for Employees (Post implementation stage). (DOCX 28 kb) Additional file 5: Topic Guide for Follow-up Focus Group. (DOC 33 kb) Additional file 6: Topic Guide for Managers (Post implementation stage). (DOCX 27 kb)

## Abbreviations

CMAI: Catering Managers Association of Ireland
FCW: food choice at work study
HR: human resources
IDA: Industrial Development Authority of Ireland
MRC: Medical Research Council
