# Supplementary material for: Barriers to and facilitators of implementing complex workplace dietary interventions: process evaluation results of a cluster controlled trial
Source: BMC Health Serv Res. 2016 Apr 21;16:139. doi: 10.1186/s12913-016-1413-7 (PMC4840486; doi:10.1186/s12913-016-1413-7)
Supplement: Additional file 1: — Topic Guide for Employees – Baseline stage. (DOCX 27 kb) [file 12913_2016_1413_MOESM1_ESM.docx]

**Topic Guide for Employees – Baseline stage**

1. **Lead in – current situation**
2. Can you give me a brief overview of your current position within this company?

- How many years have you worked here?
- How many hours a day/week do you spend at this worksite?
- How much time is allocated for lunch breaks and how do you spend this time?

1. **Health and diet**
2. Do you regard yourself as being a ‘health conscious’ person?

- Yes/no/in what way?

1. How would you describe your diet? Do you take steps to meet your dietary needs? E.g eating your 5-a-day
2. What areas could you improve on?
3. Can you tell me a bit about your diet and eating habits at work?
4. What are your favourite foods?
5. Does your diet vary with the seasons?
6. What do you drink with your meals generally?
7. Do you have regular meals or do you snack frequently?
8. **Influence on food choice at work**
9. What factors influence your food choice at work?
10. What do you think about the selection of food on offer in the canteen?
11. Do you think enough variety is provided?
12. What do you think of the quality of food available?
13. What do you think of the affordability of food in the canteen?
14. What would you eat in the canteen on a typical day?
15. What would encourage you to eat more healthily in the workplace?
16. **Previous involvement in workplace interventions/opinions on canteen food**
17. Have you ever been involved in a workplace health promotion programme either here or in a previous workplace?

- If so what did it entail?

1. What is your opinion on the food available in the canteen?
2. What is the general view among fellow employees?
3. What changes would you make to the food available if given the opportunity?
4. What kinds of meals are available?
5. Is there a wide range of choice?
6. Are low calorie options available?
7. If not, do you think healthier meals should be made available?
8. Where do you think the responsibility lies in making this a healthier workplace and ensuring that employees get nutritious and balanced meals?
9. **Expectations of the Food Choice at Work Intervention**
10. What does this study entail for you?
11. How do you feel about participating in the food study?
12. What is the general view among your colleagues?
13. What you do expect from the forthcoming programme?
14. Do you perceive any benefits to yourself?
15. Is the study a topic of conversation amongst you and your colleagues?
16. Determine if there is enthusiasm/negativity for the intervention.
17. Do you have any issues/concerns about the study?
18. **Barriers to the success of the intervention**
19. How is the study being received by your colleagues?
20. Do you think there will be any barriers to the success of the study?
21. Is there willingness for change in the workplace?
22. Do you think people will be honest in their answers?
23. What benefits (if any) do you envisage for yourself and your colleagues due to involvement in this study?
24. **Debriefing/conclusion**

Thank the interviewee for their time and effort and ask if they have any questions or anything more to add. Conclude the interview if there is no further questions and comment briefly on main findings or interesting comments which may spark further feedback. Reassure participant around issues of confidentiality, anonymity and privacy and state that findings will not reveal personal details.
