# Supplementary material for: Barriers to and facilitators of implementing complex workplace dietary interventions: process evaluation results of a cluster controlled trial
Source: BMC Health Serv Res. 2016 Apr 21;16:139. doi: 10.1186/s12913-016-1413-7 (PMC4840486; doi:10.1186/s12913-016-1413-7)
Supplement: Additional file 2: — Topic Guide for Focus Group (Baseline stage). (DOC 31 kb) [file 12913_2016_1413_MOESM2_ESM.doc]

**Topic Guide for Focus Group (Baseline stage)**

1. **Rationale and lead in**

The purpose of this focus group is to get your experiences and feelings about implementing the Food Choice at Work Study. In particular we will look at the recruitment and baseline data collection phases as these are nearly complete at all sites. We won’t be addressing the intervention directly, this is for another time. I’d like to start by going around the table and if everyone could just give their name and briefly explain what site or aspect of the study you have been involved with and when you started (recruitment/baseline collection/which sites/both).

1. **Introduction**

- Thinking about where the study is at, what’s your assessment of progress to date?
- For those who have been in contact with different sites do you notice differences between them? Can you talk more about these?
- How do you find the overall coordination within the office and between the office and the sites? That is the internal communication between the team.

1. **Recruitment process**

Ok, now I would like to start looking at more specific areas. I’d like you to think about the recruitment process to get participants to take part in the study (that is from the beginning, activities that took place on site as well as through the office).

- What has been your experience of recruiting participants?
- What was the most challenging aspect?
- What things did you experience that you feel created problems, if any, during this process? Things you find/found frustrating?
- What are the things that you feel worked well during this process? What helped?
- Were there things about recruitment that weren’t expected/ surprising?
- Do you feel there was a process in place to achieve this, a protocol from the outset? How do you feel this went?
- Were there adaptations that needed to be made? If so, how did you adapt?
- Were there noticeable differences across sites in terms of recruitment?

1. **Baseline collection stage**

Now I would like to move onto the area of the baseline data collection at the sites.

- What has been your experience of this process?
- What are the things that you feel worked well during this process? What helped?
- Were there things that, in your experience, created problems, if any, during this process?
- Were there unexpected things that cropped up during this stage?
- Any things you find/found frustrating?
- Were there adaptations that needed to be made? If so, how did that process of adaptation take place?
- Were there noticeable differences across sites?
- Any things you might suggest to improve/assist in the next phase of collection?

1. **Participants and sites**

We would have mentioned participants throughout this discussion but I’d like to spend a little time focusing on them and the sites themselves. First I want to ask about the kinds of queries/questions you get from people.

- Do you find common queries you regularly receive from participants?
- How do you deal with these or have you developed responses to these that you now use that you find effective?
- Now if we could turn to the reasons you hear from people, be it by email, on the phone or in person. I know there is an online survey that asks specifically about this but you get to talk to people and hear what the survey doesn’t capture. That is the reasons why they participated and why they don’t participate. Do people explain their reasons if they are no-shows?
- Do you imagine that an ‘out of work’ collection period might have helped?
- Do you find the people you meet with are in a hurry?
- Is this site specific? Do you think there are environmental reasons for this?
- What are your impressions of the participants you deal with?
- What’s the environment like, is it accepting of the study or is it seen as a hindrance?
- How helpful are the management on each of the sites?
- Is this view shared by management and participants or is there a divergence of views?

1. **Finishing up**

- I would like to ask if there were other issues, even those not directly related to the study, that you experienced as having an impact on the implementation of the study?
- Do you think we missed anything in the discussion?
- Does anyone have any questions at this point?

I’d like to thank you most sincerely for your time and attention here today.
