# Supplementary material for: Barriers to and facilitators of implementing complex workplace dietary interventions: process evaluation results of a cluster controlled trial
Source: BMC Health Serv Res. 2016 Apr 21;16:139. doi: 10.1186/s12913-016-1413-7 (PMC4840486; doi:10.1186/s12913-016-1413-7)
Supplement: Additional file 3: — Topic Guide for Managers (Baseline stage). (DOCX 23 kb) [file 12913_2016_1413_MOESM3_ESM.docx]

**Topic Guide for Managers (Baseline stage)**

1. **Lead In - current situation:**
2. Can you tell me about your current position with the company?

- How long have you been in this position?

- What are your weekly hours?

- What are your main responsibilities?

1. What is the management structure in the company?

**B) Health and diet**

1. How is your general overall health?

- Previous health/major illness etc.

- Establish their concept of ‘health’

1. Is your health important to you?

-Why/why not?

1. How do you manage/meet your health needs at the moment?
2. Can you tell me a little about your diet?

- Favourite food, if enjoy cooking or not
- If eat regular meals, or snack a lot
- How often eat at work and what?

1. Do you plan what/when/where you’re going to eat?
2. **Study knowledge**
3. What are your thoughts on offering healthy food choices in the workplace?

- Try and establish attitude to employees health

1. What is your impression of the food choice at work study?
2. Can you tell me how you came to be involved in the study?

- Financial/promotion/status/improving workplace health/atmosphere?
- Profile of company/company policy?

1. Can you tell me about the canteen environment at work?

- Is it liked as a place to eat?
- What influences your food choices at work?

1. **Perceptions of intervention**
2. Can you tell me a little about any previous projects you were involved in here or elsewhere?
3. What level of involvement does this company have in the study?
4. Can you tell me what this involvement involves for you directly?
5. Has this influenced your motivation for involvement?

- Level of involvement with catering stakeholders/worksite managers in terms of meetings/set up/planning of the study?
- What other things encouraged you/the company’s participation in the study?

1. Can you explain how the study will work in this company?
2. Do you have any concerns about this study?

- Barriers that may arise from success of proposed changes?
- Overall attitude to change in workplace/willingness for change?

1. What benefits do you see for your company and employees due to participation in project?
2. How do you imagine the study will be received by participants/non participants?
3. What strategies do you feel might work to promote healthy eating in the workplace?
4. What knowledge/support have you experienced so far in relation to the study?

- Role so far in intervention, future role?
- Is there enthusiasm for this intervention?

1. **Follow-up**
2. What do you/company hope to achieve from participating in the study?
3. Who do you think will benefit from this study?
4. **Debriefing**

Thank interviewee and ask if subject has anything more to add. Round off the interview if no further additions, and comment briefly on findings which may elicit further feedback. Clarify with subject if there are any additional issues they might be concerned or worried about. Reassure subject around issues of confidentially, anonymity and privacy, that findings will not reveal personal details
