# Supplementary material for: Barriers to and facilitators of implementing complex workplace dietary interventions: process evaluation results of a cluster controlled trial
Source: BMC Health Serv Res. 2016 Apr 21;16:139. doi: 10.1186/s12913-016-1413-7 (PMC4840486; doi:10.1186/s12913-016-1413-7)
Supplement: Additional file 4: — Topic Guide for Employees (Post implementation stage). (DOCX 28 kb) [file 12913_2016_1413_MOESM4_ESM.docx]

**Topic Guide for Employees (Post implementation stage)**

1. **Lead in – current situation:**
2. Can you just remind me briefly of your current position within this company?

- Have your hours changed in any way?
- Are you still working on the same shift?

1. **Influence on food choice at work:**
2. Tell me a bit about your diet and eating habits at work?

- What would you eat in the canteen on a typical day?
- What factors influence your food choice at work?

-If what’s ‘healthy’ influences their choice – prompt ‘Are there any other factors apart from considering what’s healthy and then prompt with the following:

-Length of breaks, time to eat, the physical layout of the canteen?

1. What do you think about the food on offer in the canteen?

-Enough variety provided?

-Quality?

-Affordability?

- Have you noticed any changes since the study began?

-If no – prompt on choice, portion size, more fruit and vegetables…..

- What is the general view among fellow employees?

**3)** What do you think could be done differently?

- Could meal options be healthier?
- Do you think healthier meal options should be made available?

**4)** What would you like to see?

- What changes would you make to the food if given the opportunity?

1. What would encourage you to eat more healthily in the workplace?
2. What do you think about restricting choices, perhaps things that are considered unhealthy?
3. What would you see as the role of catering/management in healthy eating in the workplace?
4. **Health and Diet:**
5. Do you do anything particular to keep healthy?

-Yes/no/what do you do?

-For example do you follow a healthy diet or exercise regime?

1. Is your diet important to you?

- Do you do anything in particular to maintain a healthy diet?

1. Have you any concerns about your health?

- Is it something you think about?

1. Have your attitudes towards health (and diet) changed since taking part in the study?

- In what way (diet), why, when did you notice a change (immediate versus gradual)
- Would you consider yourself more health conscious now?

1. **Expectations of the Food Choice at Work Intervention:**
2. What did this study involve for you?

- Why did you want to participate?
- What was the perceived benefit?

1. What do you feel worked well for you?
2. What do you feel didn’t work well for you?
3. Did you find any aspects particularly challenging?

-Did you find it difficult to get the time to attend appointments?

-How did you find the actual appointments?

-How did you find filling out the questionnaires

-Were there any negative effects?

1. **Barriers to the Study:**
2. Do you think there were any barriers to the study?

-Some people mentioned lack of interest/difficulty in breaking old habits; do you think these barriers existed?

**2)** In earlier interviews some people felt the intervention should have been targeted at certain groups?

-What do you think?

-Would you agree or disagree?

**3)** Do you think anything should have been done differently?

-Invitation, communication, assessments, types of changes,

**Debriefing/conclusion**

1. Thank the interviewee for their time and effort and ask if they have any questions or anything more to add.
2. Conclude the interview if there is no further questions and comment briefly on main findings or interesting comments which may spark further feedback.
3. Reassure participant around issues of confidentiality, anonymity and privacy and state that findings will not reveal personal details.
