# Supplementary material for: Barriers to and facilitators of implementing complex workplace dietary interventions: process evaluation results of a cluster controlled trial
Source: BMC Health Serv Res. 2016 Apr 21;16:139. doi: 10.1186/s12913-016-1413-7 (PMC4840486; doi:10.1186/s12913-016-1413-7)
Supplement: Additional file 5: — Topic Guide for Follow-up Focus Group. (DOC 33 kb) [file 12913_2016_1413_MOESM5_ESM.doc]

**Topic Guide for Follow-up Focus Group**

**Question 1:** Thinking about where the study is at, what is your assessment of progress to date?

- For those who have been in contact with different sites do you notice differences between them? Can you talk more about these?
- How do you find the overall coordination within the office and between the office and the sites? That is the internal communication between the team.

*Intervention Phase*

**Question 2:** I would like to start looking at more specific areas. I’d like you to think about the intervention that happened at each site, understanding that each site had a different level of intervention.

- What has been your experience of conducting the intervention onsite?
- What has been your experience of conducting the intervention overall?
- What was the most challenging aspect?
- What things did you experience that you feel created problems, if any, during this process?
- What are the things that you feel worked well during this process? What helped?
- Were there things about this phase that weren’t expected/ surprising?
- Do you feel there was a process in place to achieve this, a protocol from the outset? How do you feel this went?
- Were there adaptations that needed to be made? If so, how did you adapt?
- How did the different sites respond to the intervention?
- What kind of comments did you receive from participants?

*Follow-up collection stage*

**Question 3:**

Now I would like to move onto the area of the follow-up data collection at the sites.

- What has been your experience of this process?
- How easy/difficult was it to get participants to come back at this stage?
- What are the things that you feel worked well during this process? What helped?
- Were there things that, in your experience, created problems, if any, during this process?
- Were there unexpected things that cropped up during this stage?
- What feedback did you receive from the participants at this point?
- Any things you find/found frustrating?
- Was this plan implemented accordingly?
- Were there adaptations that needed to be made? If so, how did that process of adaptation take place?
- Were there noticeable differences across sites?
- Any things you might suggest to improve/assist in the next phase of collection/ suggestions for others carrying out a similar study?

*Participants and Sites*

**Question 4:**

We would have mentioned participants throughout this discussion but I’d like to spend a little time focusing on them and the sites themselves. First I want to ask about the kinds of queries/questions you get from people.

- How did the respondents feel about their participation in the study?
- Was there any talk by the participants about the management's role in this study?
- Now if we could turn to the reasons you hear from people, be it by email, on the phone or in person. Do people explain their reasons if they do not want to continue with the study?
- What are your impressions of the participants you deal with?
- What’s the environment like, is it accepting of the study or is it seen as a hindrance?
- How helpful are the management on each of the sites?
- Is this view shared by management and participants or is there a divergence of views?

*Finishing Up*

**Question 5:**

I would like to ask if there were other issues, even those not directly related to the study, that you experienced as having an impact on the implementation of the study?

- Do you think we missed anything in the discussion?
- Does anyone have any questions at this point?
