# Supplementary material for: Barriers to and facilitators of implementing complex workplace dietary interventions: process evaluation results of a cluster controlled trial
Source: BMC Health Serv Res. 2016 Apr 21;16:139. doi: 10.1186/s12913-016-1413-7 (PMC4840486; doi:10.1186/s12913-016-1413-7)
Supplement: Additional file 6: — Topic Guide for Managers (Post implementation stage). (DOCX 27 kb) [file 12913_2016_1413_MOESM6_ESM.docx]

**Topic Guide for Managers (Post implementation stage)**

1. **Lead in – current situation:**
2. Can you just remind me briefly of your current role within this company?

-What was your involvement in the study?

-Has this changed over the course of the study?

**2)** What encouraged you/the company’s participation in the study?

**3)** Do ye do anything to promote health already?

1. What did you as 1) a manager and 2) as a company hope to achieve by participating in this study?
2. **Study Knowledge**
3. What has been your overall impression of the food choice at work study?

- How did the study work here?

-What did UCC do?

- Who was involved in setting it up?

-After UCC came and met with ye, what did ye have to do then in order to get this on board?

- How was the study received here?

2) Can you tell me about the canteen environment at work now?

- How is the canteen viewed within the workplace?
- Have you noticed any changes since the study began, if so what were they?

-food choice, portion size, increase in fruit and vegetables….

- Also what do you think influences employees food choices at work?

-Time, physical layout of canteen, cost, break times?

1. ***Health and Diet (Only include if the manager is participating in the study)***
2. Do you do anything particular to keep healthy?

-Yes/no/what do you do?

-For example do you follow a healthy diet or exercise regime?

1. Is your diet important to you?

- Do you do anything in particular to maintain a healthy diet?

1. Have you any concerns about your health?

- Is it something you think about?

1. Have your attitudes towards health (and diet) changed since taking part in the study?

- In what way (diet), why, when did you notice a change (immediate versus gradual)
- Would you consider yourself more health conscious now?

1. **Perceptions of the intervention**
2. How do you feel the study was received by participants and secondly non participants?
   - Any resistance, why?
3. Did you have any concerns about this study before it started?

- Did you think it would have any negative impacts?
- Were there any barriers to changes?
- Overall what was the attitude to change in the workplace/

-Was there willingness for change?

- Did any of these issues transpire?

**3)** What were the benefits of participating?

**4)** Who do you think benefited from this study?

- The Company?
- The employees?

**5)** What aspects do you think worked well? Not so well?

**6)** Is there anything you would do different?

**7)** Are there any other strategies that you feel might work well in order to promote healthy eating in the workplace?

- What do you think about restricting choices, perhaps things that are considered unhealthy?
- was there any backlash from employees (site c)
- or would you think there would be any backlash (site b)
- What do you think about nutrition education?
- How do you feel the group nutrition sessions, posters, traffic light labelling were perceived by employees?

**8)** We know that there’s an alarming prevalence of type 2 diabetes, CVD and obesity in Ireland. We also know that we can improve employee’s long term dietary behaviours with effective workplace health promotion, what you would you then see as the role of catering/management in healthy eating in the workplace?

- Where do you think it should start?

**9)** How do you think employees perceive the employer trying to make an effort?

**10)** Do you have any suggestions for researchers carry out a study like this in future?

**11)** Would you participate again? If not, why not?

**Debriefing/conclusion**

1. Thank the interviewee for their time and effort and ask if they have any questions or anything more to add.
2. Conclude the interview if there is no further questions and comment briefly on main findings or interesting comments which may spark further feedback.
3. Reassure participant around issues of confidentiality, anonymity and privacy and state that findings will not reveal personal details.
